# Analyzing microtomography data with Python and the scikit-image library

**DOI:** 10.1186/s40679-016-0031-0

**Published:** 2016-12-07

**Authors:** Emmanuelle Gouillart, Juan Nunez-Iglesias, Stéfan van der Walt

**Affiliations:** 1 Surface du Verre et Interfaces, UMR 125 CNRS/Saint-Gobain, 93303 Aubervilliers, France; 2 Victorian Life Sciences Computation Initiative, University of Melbourne, Carlton, VIC Australia; 3 Division of Applied Mathematics, Stellenbosch University, Stellenbosch, South Africa

**Keywords:** Scikit-image, Python, Image processing library, 3D image

## Abstract

The exploration and processing of images is a vital aspect of the scientific workflows of many X-ray imaging modalities. Users require tools that combine interactivity, versatility, and performance. scikit-image is an open-source image processing toolkit for the Python language that supports a large variety of file formats and is compatible with 2D and 3D images. The toolkit exposes a simple programming interface, with thematic modules grouping functions according to their purpose, such as image restoration, segmentation, and measurements. scikit-image users benefit from a rich scientific Python ecosystem that contains many powerful libraries for tasks such as visualization or machine learning. scikit-image combines a gentle learning curve, versatile image processing capabilities, and the scalable performance required for the high-throughput analysis of X-ray imaging data.

## Background

The acquisition time of synchrotron tomography images has decreased dramatically over the last decade, from hours to seconds [29]. New modalities such as single-bunch imaging provide a time resolution down to the nanosecond for radiography [41]. However, the time subsequently spent in processing the images has not decreased as much, so that the outcome of a successful synchrotron imaging run often takes weeks or even months to be transformed into scientific results.

Transforming billions of pixels and voxels to a few meaningful figures represents a tremendous data reduction. Often, the sequence of operations needed to produce these data is not known beforehand, or might be altered due to artifacts [31], or to an unforeseen evolution of the sample. Image processing necessarily involves trial and error phases to choose the processing workflow. Therefore, image processing tools need to offer at the same time enough flexibility of use, a variety of algorithms, and efficient implementations to allow for fast iterations while adjusting the workflow.

Several software applications and libraries are available to synchrotron users to process their images. ImageJ [1, 48] and its distribution Fiji [45] is a popular general-purpose tool for 2D and 3D images, thanks to its intuitive menus and graphical tools, and the wealth of plugins contributed by a vivid community [46]. Software specialized in analyzing synchrotron data is available as well, such as XRDUA [17] for diffraction images obtained in powder diffraction analysis, or for 3D images, commercial tools such as Avizo 3D software (TM), or ToolIP/MAVIkit [19] are appreciated for an intuitive graphical pipeline and advanced 3D visualization. Some synchrotrons have even developed their own tools for volume processing, such as Pore3D [9] at the Elettra facility. Alternatively, the use of a programming language gives finer control, better reproducibility, and more complex analysis possibilities, provided classical processing algorithms can be called from libraries—thereby limiting the complexity of the programming task and the risk of bugs. Matlab (TM) and its image processing toolbox are popular in the academic community of computer vision and image processing. The Python language is widely used in the scientific world and in synchrotron facilities. As a general-purpose language, Python is used in synchrotrons to control device servers [8, 15, 51], to access raw data of X-ray detectors [27], to reconstruct tomography volumes from radiographs [23, 32], and in data processing packages for macromolecular crystallography [3], azimuthal integration of diffraction data [4], or fluorescence analysis [50, 52].

scikit-image [54] is a general-purpose image processing library for the Python language, and a component of the ecosystem of Python scientific modules commonly known as Scientific Python [34]. Like the rest of the ecosystem, scikit-image is released under a permissive open-source license and is available free of charge. Most of scikit-image is compatible with both 2D and 3D images, so that it can be used for a large number of imaging modalities, such as microscopy, radiography, or tomography. In this article, we explain how scikit-image can be used for processing data acquired in X-ray imaging experiments, with a focus on microtomography 3D images. This article does not intend to be a pedagogical tutorial on scikit-image for X-ray imaging, but rather to explain the rationale behind the package, and provide various examples of its capabilities.

## Overview and first steps

In this section, we provide a short overview of the typical use patterns of scikit-image, illustrated by short snippets of code. Since Python is a programming language, the user interacts with data objects and images through code, which is either entered and executed in an interactive interpreter, or written in text files (so-called scripts) that are executed.

**Fig. 1:**
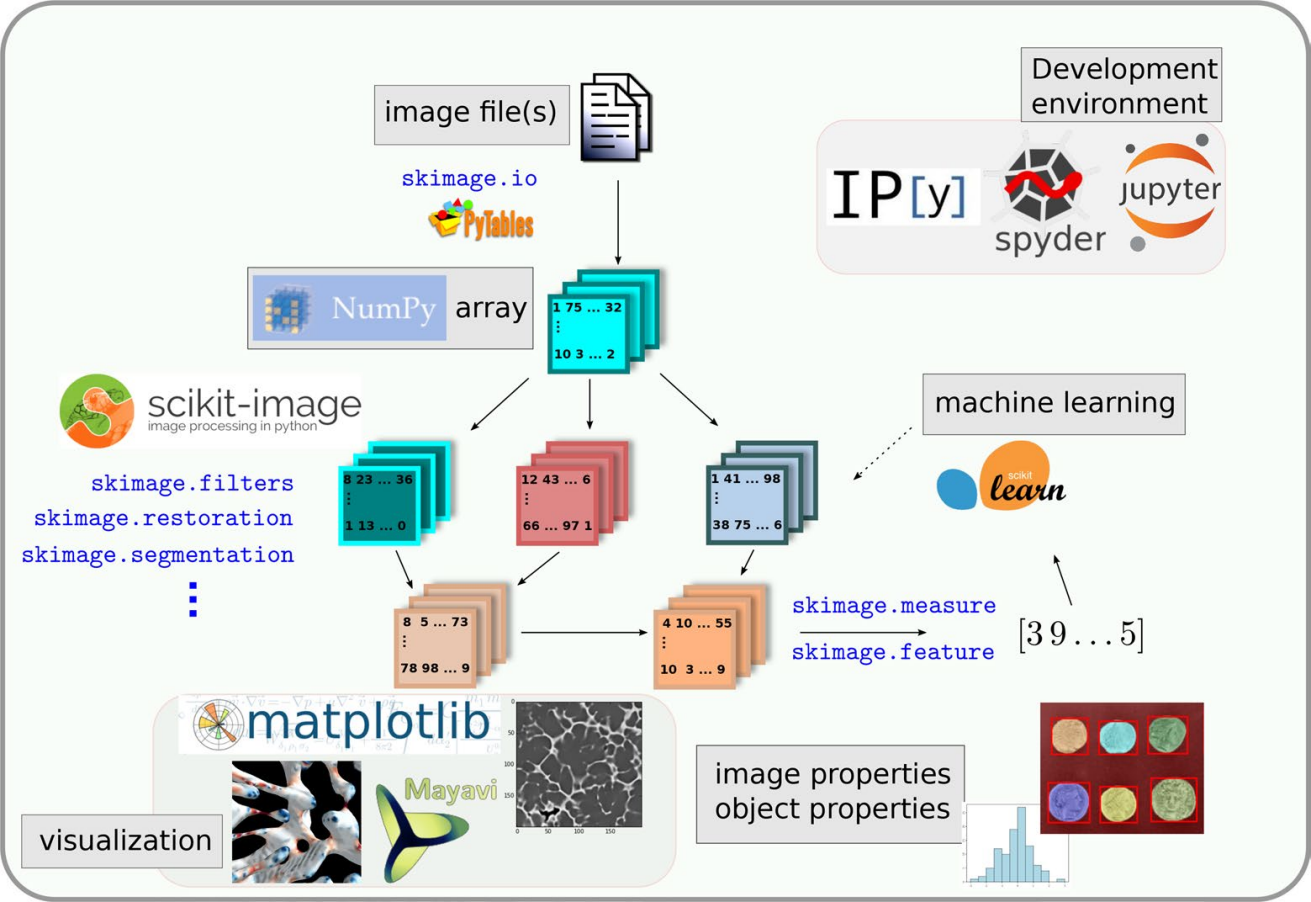
scikit-image and the Scientific Python ecosystem. Images are opened from files as NumPy arrays. Functions of scikit-image transform image arrays into other arrays with the same dimensions, or into arrays of numbers corresponding to features of the image. The output of scikit-image functions can be passed to other Python modules relying on NumPy arrays, such as SciPy or scikit-learn. Image-shaped arrays are transformed into visualizations with matplotlib (2D) or Mayavi (3D). A variety of environments is available for code development and execution, from classical IDEs to Jupyter notebooks

Images are manipulated as numerical arrays, each with a single, uniform data type. This common format guarantees interoperability with other libraries and straightforward access to and interpretation of computer memory. The N-dimensional (2D, 3D, ...) numerical array object is provided by the NumPy module [53].

In image processing in Python, one of the first tasks then is to generate NumPy arrays, which is often achieved by reading data from files. We read one 2-dimensional image from a file and display it as follows:
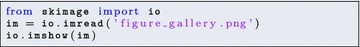


skimage is the name under which scikit-image is imported in Python code. Note that functions (such as imread that reads an image file, or imshow that displays an image) are found in thematic submodules of skimage, such as io for Input/Output.

A stack of 2D images, such as tomography slices generated by a reconstruction algorithm, can be opened as an image collection or a 3D array: 
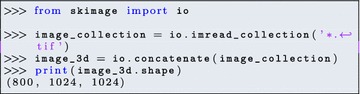


Raw data formats can be opened using the NumPy functions fromfile (to load the array into memory) or memmap (to keep the array on disk). The following code creates an array from a raw image file of unsigned 16-bit integers with a header of 1024 bytes: 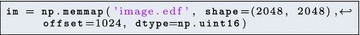 For every raw data specification, it is thus very easy to write a reader using np.memmap (see for example https://github.com/jni/python-redshirt). hdf5 files are accessed using modules such as h5py, pytables.

scikit-image has a simple Application Programming Interface (API), based almost exclusively on functions. Most functions take an image (i.e., a multi-dimensional array) as an input parameter: 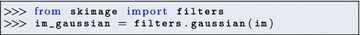


Optional parameters can be passed as Python *keyword arguments*, in addition to the image parameter. 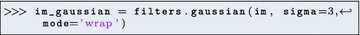 A few functions require several arrays to be passed, such as the watershed segmentation algorithm that takes as parameters the image to be segmented, and an image of markers from which labels are propagated: 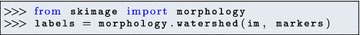 Therefore, the image processing workflow can be seen as a directed graph (a richer structure than a linear pipeline), where nodes are image-shaped arrays, and edges are functions of scikit-image transforming the arrays (see Fig. 1).

Most functions transparently handle 2D, 3D, or even higher-dimensional images as arguments, so the same functions can be used to process tomography, microscopy, or natural images. The rest raise an error when passed a 3D argument: 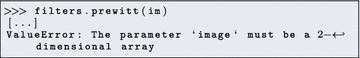 However, the proportion of functions supporting 3D images is always increasing, thanks to the many contributors to the library.

While the majority of functions return processed images, returns can also be numerical value(s) such as pixel coordinates of objects of interest or statistical information about the image: 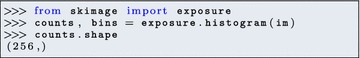


## The Python ecosystem

The benefits of scikit-image for image processing come not only from the features of the package alone, but also from the rich environment surrounding scientific Python [34, 38]. Figure 1 illustrates how several components of this ecosystem combine into a sophisticated image processing workflow.

*NumPy arrays* are the cornerstone of the Scientific Python ecosystem, and of scikit-image operations in particular. NumPy “one-liners” include cropping or downsampling an image, or retrieving pixels corresponding to a given label in a segmentation. To illustrate the compactness of NumPy code, consider modifying pixel values below a threshold. This operation can be written as
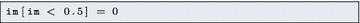
exploiting the ability to index arrays with boolean arrays, also called *masking*. NumPy uses memory sparingly and avoids making new copies of arrays whenever possible, an important requirement when dealing with the gigabyte-sized images of tomography. For example, cropping a subvolume as follows does not create a copy of the original array
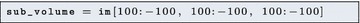
but instead refers to the correct memory offsets in the original.

**Fig. 2:**
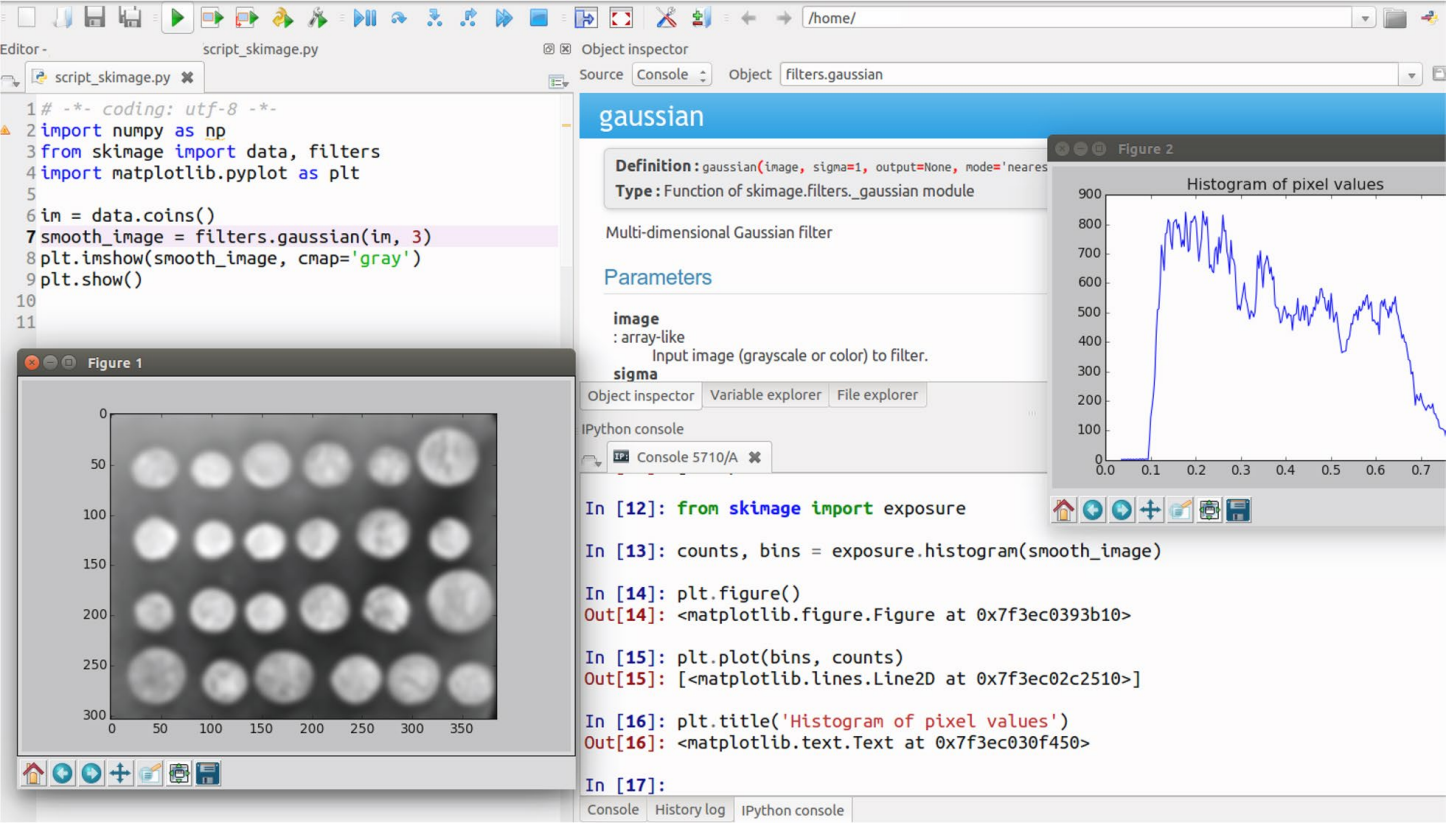
The Spyder IDE integrates a text editor (with syntax highlighting), the IPython interpreter, as well as a panel for code introspection (online help, variable explorer, ...)

*Interpreter and development environment* While several interpreters are available to execute Python instructions and scripts interactively, the most popular in the scientific world is IPython [37, 44]. IPython is an advanced interpreter, which integrates syntax highlighting, text auto-completion, a debugger, introspection and profiling methods, and online help. Several integrated development environments (IDEs) come bundled with IPython, together with other components such as a text editor. Notable examples include Spyder (Fig. 2), PyCharm, and Visual Studio Code.

**Fig. 3:**
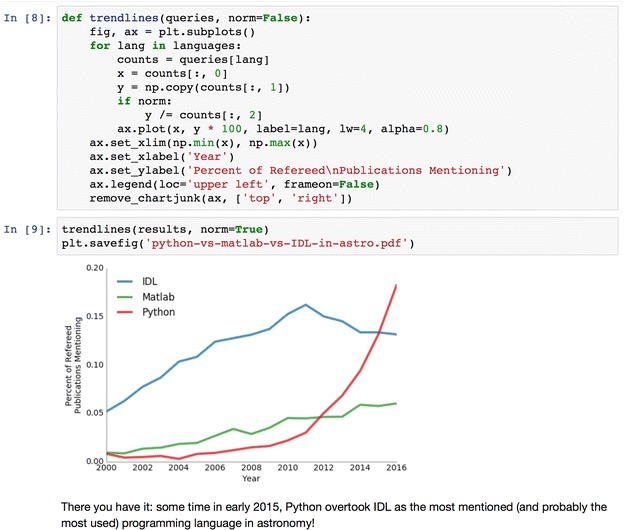
The Jupyter notebook allows mixing of computer code (*top*), plot and text output (*middle*), and free-form narrative text (*bottom*). This makes it ideal to record and report code-based analyses. Screenshot from [33]

The Jupyter notebook [26] is a web application that grew out of the IPython project. Jupyter notebooks provide an interactive development environment within a web browser, where live code can be enriched by explanatory text, equations, and visualizations (Fig. 3). Jupyter notebooks render directly as webpages on GitHub, making them a straightforward tool to publish online a script and its output. As of July 2016, more than 500,000 Jupyter notebooks were posted on GitHub, demonstrating their wide adoption by the community as workflow-sharing tools (http://archive.ipython.org/media/SciPy2016JupyterLab.pdf).

**Fig. 4:**
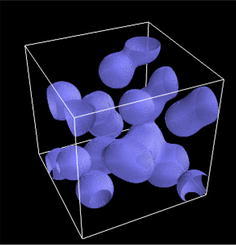
Simple 3D visualization realized with Mayavi

*Visualization libraries* Visualizing images is an important component of the image processing workflow, used to inspect the final result and to adjust the parameters of intermediate processing operations. matplotlib [24] is the most popular 2D plotting library of the Python ecosystem. It can be used to visualize 2D data such as color or grayscale images, and 1D data such as contour lines, outlines of segmented regions, histograms of gray levels. Although matplotlib has simple 3D plotting capabilities, we recommend using the mayavi module [42] for applications requiring advanced 3D visualization, such as tomography. mayavi is based on the VTK toolkit. It exposes a simple API for visualizing data passed as numpy arrays. For example, visualizing the surface of binary data can be written as
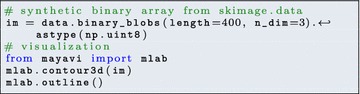
(see Fig. 4 for the resulting visualization).

For more advanced visualizations, a large majority of VTK capabilities can be accessed through mayavi’s pipeline API. mayavi offers a good trade-off between simplicity of use for common operations, and accessibility to more sophisticated capabilities such as responsive visualizations.

*Advanced toolkits for signal processing and data science* scikit-image is only one Python module that can be used for data processing, among many others. A very popular module is scikit-learn [36], a Python module for machine learning using NumPy arrays. Local features of an image (such as local statistics of gray levels, or geometric points of interest) or features of segmented objects (e.g., geometrical and intensity characteristics of segmented particles) can be extracted with functions from skimage.feature (see Fig. 1). It is then possible to use a *classification* algorithm from scikit-learn to label pixels (a segmentation task) or to classify whole images or objects that have already been segmented. The near-universal use of NumPy arrays ensures the interoperability between these packages, so that just a few lines of code are sufficient to create these sophisticated workflows.

The modularity of the Scientific Python ecosystem may be confusing at first sight, but the core modules of this ecosystem are almost perfectly compatible, thanks to the shared use of NumPy arrays and common development practices (although they are developed in parallel by different teams). Several “distributions,” such as Anaconda or Canopy, bundle together the most popular libraries, including scikit-image.

### Image processing capabilities

**Fig. 5:**
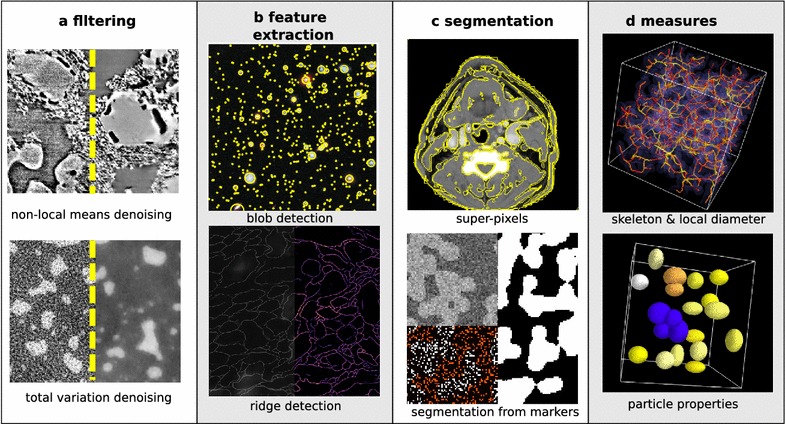
Typical image processing operations with **scikit-image**. Data are synthetic, unless stated otherwise. **a** Filtering−*Top* non-local means denoising of an image with a fine-grained texture, acquired by in situ synchrotron microtomography during glass melting [21]. *Bottom* total-variation denoising of an image with two phases, corresponding to phase-separating silicate melts observed by in situ tomography [7]. **b** Feature extraction−*Top* Hubble deep field (NASA, public domain), blob detection using the Laplacian of Gaussian method. *Bottom* ridge detection using the leading eigenvalue of the Hessian matrix, neuron image from CREMI challenge (https://cremi.org/data/). **c** Segmentation—*Top* super-pixel segmentation of a CT slice of the human head [13], using Felzenszwalb’s algorithm [18]. *Bottom* random walker segmentation (*right*) of noisy image (*top-left corner*), using histogram-determined markers (*bottom-left corner*). **d** Measures—*Top* visualization of local diameter (color-coded on the skeleton curve) of an interconnected phase (represented in *violet*). *Bottom* particles color-coded according to their extent

*Capabilities* scikit-image offers most classical image processing operations, such as exposure and color adjustment, filtering, segmentation, feature extraction, geometric transformations, and measurements of region characteristics. In addition to common operations, some advanced algorithms are also implemented; a selection of which is illustrated in Fig. 5. In the following, we briefly illustrate how scikit-image can be used for some typical image processing tasks encountered when analyzing tomographic images: denoising, mid-range feature detection, segmentation, and measurement of region properties. For the sake of brevity, other tasks such as contrast manipulation or geometric transformations are not described here; the interested reader is referred to the documentation of scikit-image.

Tomographic images often suffer from artifacts or poor signal-to-noise ratio. Therefore, *denoising* data is often the first step of an image processing workflow. Several denoising filters are available for restoring these images, ranging from general-purpose median and bilateral filters to those more suited to specific applications. For example, total-variation denoising [12, 20] is ideal for restoring piecewise-constant images (see Fig. 5a), such as images with a small number of phases encountered in materials science [7]. Conversely, images with a fine-grained texture are better preserved with non-local means denoising, a patch-based algorithm [10] (see Fig. 5a).

Detecting the presence of objects or extracting pixels corresponding to objects (a task known as segmentation) is an important task of image analysis for medical or materials science applications. scikit-image offers a wide variety of functions for *detecting geometrical features* of interest in an image. In order to detect thin boundaries, the ridges of an image can be identified as regions for which the leading eigenvalue of the local Hessian matrix is high (see Fig. 5b). In the Fourier space, peaks in 2D Bragg diffraction patterns can be extracted using blob detection methods [4], such as the Laplacian of Gaussian method (see Fig. 5b).

*Segmentation* of regions of interest can be achieved using one of the various strategies, depending on the characteristics of the image. Images with a clear contrast between regions can be segmented automatically, thanks to several thresholding algorithms, including an adaptive local thresholding algorithm aimed at images with contrast variations. Super-pixel algorithms [2, 18] create an over-segmentation of images in super-pixels, by grouping pixels that are close together both in color- and spatial distance (see Fig. 5c). Region-growing algorithms, such as the morphological watershed or the random walker [22], propagate the labels of user-defined markers through the image (see Fig. 5c). The active contour algorithm [25] fits snake contours to features of the image, such as edges or high-brightness regions.

Following segmentation, the characteristics of labeled regions (particles, porosities, organs, …) resulting from a segmentation can be measured using the measure submodule. The different connected components (e.g., bubbles or non-touching particles) of a binary image are labeled with the measure.label function. Properties of labeled regions such as size, extent, center of mass, or mean intensity value are accessed with measure.regionprops (see Fig. 5d). Local characteristics of a region can be retrieved as well: Fig. 5d shows how the local diameter of open porosity is measured by combining a skeletonization of the porosity channels, and the distance transforms to the other phase measured on the skeleton.

*Performance* Given the large size of tomography datasets, the execution speed of image processing operations is of critical concern. scikit-image relies mostly on calls to NumPy operations, of which most are performed in optimized compiled code (C or Fortran). Performance-critical parts of scikit-image that cannot call efficient NumPy code are implemented in Cython. Cython [5] is an extension of the Python language that supports explicit type declarations, and is compiled directly to C. Therefore, the performance of scikit-image can be close to the one of the libraries written in a compiled language such as C++ or Java. For example, computing the watershed segmentation of a 2000 × 2000 array of floats into 1000 regions took about 1 s using 1 CPU of an off-the-shelf laptop, and 10 s for a 256 × 256 × 256 image segmented into 2000 regions. Similar timescales were obtained with mahotas, a Python package implemented exclusively in C++ [14] (with a slight advantage for mahotas).

However, basic scikit-image code runs on a single core. Computing workstations and servers used for X-ray imaging typically have several tens of cores. Parallelization of the computing workflow can be achieved in multiple ways. The most trivial parallelization scheme consists of applying the same workflow to different images, on different cores. However, finer-grained parallelization is preferable when prototyping the processing workflow.

An easy solution consists in dividing an image into smaller images (with or without overlap, depending on the operation), and to apply the same operation on the different sub-images, on different cores. Creating overlapping chunks is easy with the dedicated function view_as_windows (or view_as_blocks for contiguous non-overlapping chunks): 
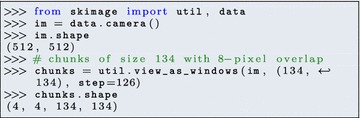
 The joblib library enables easy parallel processing. Looping over the different blocks, and dispatching the computation over several cores, is realized with the following syntax: 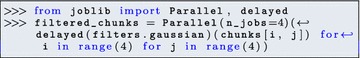 scikit-image also offers experimental support for a more integrated parallel processing pipeline, thanks to the dask [43] module: 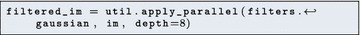 The size of chunks is determined automatically from the number of available cpus, or can be specified by the user.

*Caching* provides another tool to speed up data analysis. A situation that often arises is that, while prototyping a workflow, scripts (containing the image processing pipeline) are run several times to experiment with parameters. joblib provides a caching mechanism that avoids the repetition of function calls, if their arguments have not changed: 
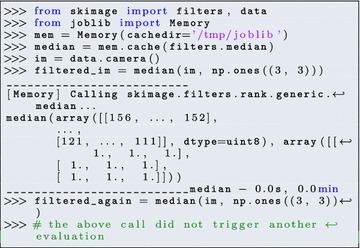


Finally, we note that the biggest performance improvements often come from the improvement of algorithms (as opposed to computing architectures only). For example, non-local means denoising [10] is a costly operation, since it requires several nested loops, on all pixels and on neighboring patches to be compared with the pixel-centered patch. Thanks to the implementation of a more recent algorithm [16] that modifies the internal organization of loops, it was possible to improve the execution time by a factor of roughly ten times. The large size of the scikit-image community makes it likely for algorithmic improvements to be discussed regularly. During the code review process, a close watch is also kept on memory consumption, since for large image sizes, transfers between computer memory (RAM) and CPU cache are often a serious performance bottleneck.

## Documentation

The quality of software documentation is (perhaps especially) important in software aimed at scientists. scikit-image users have access to several kinds of documentation. All functions are documented using the NumPy documentation standard [35], which is universal across all major Scientific Python packages. The standard includes a description of all input and output variables and their data types, together with explanations of what each function does and how to use it. Function documentation is accessible online or within the development environment itself (IPython, Spyder, Jupyter Notebook...).

**Fig. 6:**
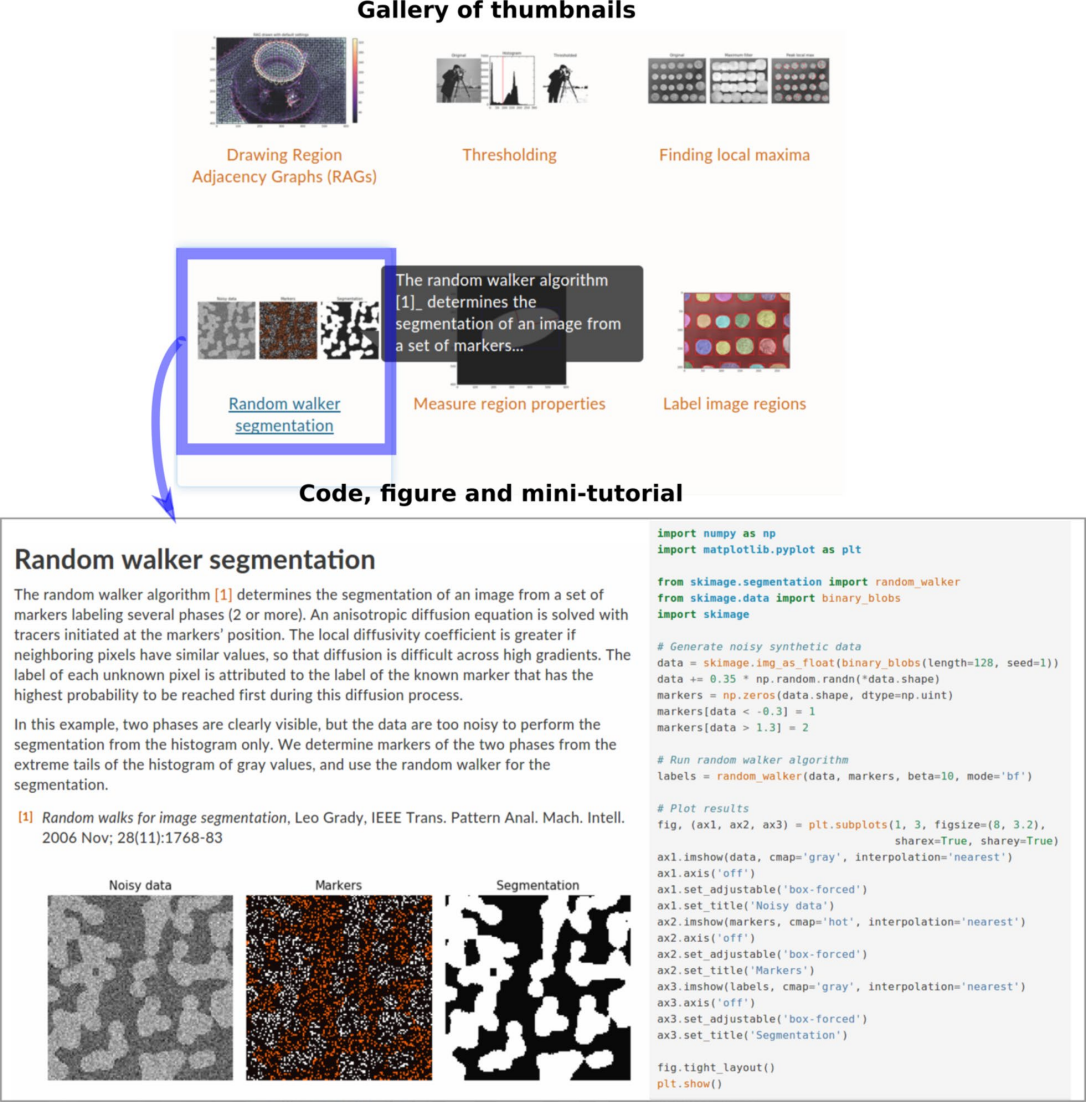
Gallery of examples of **scikit-image**. The gallery of examples consists of an array of thumbnails (*left*), which link to example webpages, each centered on a specific image processing task. Each webpage includes Python code generating a figure, the figure itself, and a short tutorial explaining the image processing operations and the code

In addition, a graphical gallery of examples (http://scikit-image.org/docs/dev/auto_examples/), part of which is displayed in Fig. 6, showcases graphical examples of common image processing operations. The examples are organized as an array of thumbnails with a short title (see Fig. 6 left). These thumbnails link to the webpage of the corresponding example, which features a mini-tutorial on the image processing method, the code needed to run the example, and the figure generated by the example. Since the graphical gallery is an efficient way to inform users about the features of scikit-image, every new feature integrated in the package must include an example for the gallery. Longer tutorials and a more narrative documentation is available as well in the online User Guide of scikit-image. The User Guide explains in particular “big picture,” foundational aspects of scikit-image, such as its use of NumPy arrays as images, or how the package interacts with other parts of the scientific Python ecosystem.

Finally, tutorials on scikit-image are available in various places, either as YouTube videos, or in the SciPy Lecture Notes [55], a comprehensive online book of Scientific Python tutorials.

## Development and use of scikit-image

*Who uses scikit-image* Estimating the number of active users of an open-source package is a difficult task. Download statistics, for example, largely overestimate the number of active users, all the more if the package is bundled with others in a software distribution, such as Anaconda or Canopy. A view closer to reality can be obtained by analyzing the statistics of visits of the online help, available on the project website. As of the first half of 2016, 20,000 unique visitors visited the scikit-image website every month at http://scikit-image.org/, from 138 countries.

The scikit-image paper of 2014 [54] has been cited by 120 research works (as of August 2016, according to Google Scholar), among which are studies that used X-ray imaging in fields such as medical imaging [6, 30, 49], materials science [7], or geoscience [47].

*Development process* scikit-image is developed by a diverse team of volunteers. More than 170 individuals have contributed to the package. The large number of developers and users is key to project’s sustainability. The development process takes place on GitHub https://github.com/scikit-image/scikit-image, where users and developers propose and discuss new contributions, report bugs, or submit ideas for improvements. A release cycle of one or two releases every year ensures that new features are propagated to users on a regular basis.

## Discussion—current limitations and challenges

While we emphasize the assets of scikit-image for processing X-ray images, one should be aware of current limitations.

*Speed of execution* Although scikit-image approaches the speed of execution of compiled (C++, Java) code, it cannot reach the performance of code optimized for the GPU, or the hand-tuned CPU-specific optimizations found in OpenCV [40]. At the moment, scikit-image is not the best tool for ultrafast computations where the workflow is known beforehand, simple, and stable. However, it is an excellent tool for exploring image data interactively and testing different algorithms—an important component of data processing in scientific work, and its speed of execution is sufficient for processing gigabyte-sized tomographic images in seconds to minutes. Moreover, the multiprocessing capability of scikit-image is likely to improve in the near future.

*3D compatibility* Currently, about two-thirds of scikit-image functions transparently handle 2D or 3D arrays, with the remainder limited to 2D analysis, often unnecessarily. Improved support for 3D and higher-dimensional volumes is on the project roadmap.

*Documentation for domain-specific applications* Some image processing libraries or applications address a specific scientific domain, such as CellProfiler [11, 28] for biological images. The documentation of such projects often showcases examples that are close to the experience of the targeted community. Since scikit-image is application-agnostic, applications such as tomographic imaging are not mentioned in detail in the documentation of scikit-image. A possible improvement would be to write comprehensive tutorials addressing specific communities, and to refer to these tutorials from the main scikit-image documentation.

## Getting started

Scientists interested in experimenting with scikit-image are invited to read the installation instructions at http://scikit-image.org/docs/stable/install.html. scikit-image can be installed either bundled in a Scientific Python distribution, such as Anaconda (conda command line) or Canopy, or stand alone (along with its dependencies) using a installer/packager such as pip or Ubuntu’s Aptitude.

The Getting Started section of the online User Guide (http://scikit-image.org/docs/dev/user_guide/getting_started.html) provides a good launch pad for beginners, and gently leads into other sections of the user guide. A gallery of examples (http://scikit-image.org/docs/dev/auto_examples/) lets users find applications close to their needs. Although most examples in the gallery use 2D images, many are applicable to 3D images as well.

Assistance on matters not covered by the documentation is provided on the dedicated mailing-list scikit-image@googlegroups.com or on Stack Overflow http://stackoverflow.com/questions/tagged/scikit-image.

## Conclusions

scikit-image offers a wide variety of image processing algorithms, using a simple interface natively compatible with 2D and 3D images. It is well integrated into the Scientific Python ecosystem, so that it interfaces well with visualization libraries and other data processing packages. scikit-image has seen tremendous growth since its creation in 2009, both in terms of users and included features. In addition to the growing number of scientific teams that use scikit-image for processing images of various X-ray modalities, domain-specific tools are now using scikit-image as a dependency to build upon. Examples include tomopy [23] for tomographic reconstruction or DIOPTAS [39] for the reduction and exploration of X-ray diffraction data. It is likely that more application-specific software will benefit from depending on scikit-image in the future, since scikit-image strives to be domain-agnostic and to keep the function interface stable. On the end-user side, future work includes better integration of parallel processing capabilities, completion of full 3D compatibility, an enriched narrative documentation, speed enhancements, and expansion of the set of supported algorithms.
